# Does postcholecystectomy increase the risk of colorectal cancer?

**DOI:** 10.3389/fmicb.2023.1194419

**Published:** 2023-06-22

**Authors:** Zhenyu Dong, Ruixian Shi, Pengda Li, Xiaobiao Song, Fan Dong, Jianmin Zhu, Riga Wu, Zhi Liang, Mingyue Du, Jijun Wang, Zhigang Yang

**Affiliations:** ^1^Department of General Surgery, Baotou Central Hospital, Baotou, Inner Mongolia, China; ^2^Baotou Medical College, Baotou, Inner Mongolia, China; ^3^Department of Neurology, Baotou Central Hospital, Baotou, Inner Mongolia, China; ^4^Inner Mongolia Medical University, Hohhot, Inner Mongolia, China; ^5^Department of General Surgery, The Second Affiliated Hospital of Baotou Medical College, Baotou, Inner Mongolia, China; ^6^Department of Urology, Baotou Central Hospital, Baotou, Inner Mongolia, China

**Keywords:** cholecystectomy, colorectal cancer, bile acid, intestinal flora, microbiome

## Abstract

With the increasing number of cholecystectomy and the high proportion of colorectal cancer in malignant tumors, the question of whether cholecystectomy is a risk factor for colorectal disease has been widely concerned. After reviewing the literature at home and abroad, the authors will summarize the research progress of the correlation between the occurrence of colorectal tumors after cholecystectomy, in order to provide help for the prevention and treatment of colorectal tumors.

## 1. Introduction

The gallbladder is one of the most important organs of the digestive system, which has the functions of movement, transport, absorption, and secretion and can concentrate and store bile between meals. After eating food, the gallbladder in the human body drains bile into the intestinal cavity through its own contraction, which can not only regulate the enterohepatic circulation of bile acids but also promote the absorption of dietary lipids (Qi et al., 2019). During fasting, liver bile continues to flow into the gallbladder cavity, where it is concentrated and acidified before being stored in the gallbladder (Housset et al., 2016). The movement function of the gallbladder has a great influence on the metabolism and enterohepatic circulation of the bile acid. Around the world, gallstones are one of the most common digestive disorders, with a worldwide prevalence of 10–15% (Shabanzadeh, 2018). In recent years, the prevalence of gallstones has gradually increased due to the increase in high-calorie, high-carbohydrate, and low-fiber diets and the decrease in physical activity (Tsai et al., 2008). Although most affected individuals remain asymptomatic during their lifetime, 10–25% will develop symptomatic gallstone disease, the consequences of which can range from simple biliary colic to potentially life-threatening complications such as acute or chronic cholecystitis, choledocholithiasis, cholangitis, biliary pancreatitis, and rarely, gallbladder cancer (Sakorafas et al., 2007). Currently, cholecystectomy is internationally recognized as the best treatment for symptomatic gallstones. Cholecystectomy can be performed in two main ways as follows: a laparoscopic or a classic open operation technique. Compared with the classic way, laparoscopic cholecystectomy is a relatively minimally invasive surgical procedure and has basically replaced the open technique for cholecystectomies since the early 1990s (Chattopadhyay and Das, 2020). Several advantages have made it a popular procedure over the past few decades, including a short hospital stay, quick return to normal activities, reduced pain after surgery, more acceptable cosmetic results, less morbidity, and less mortality (Islam et al., 2021). With the emergence of laparoscopic technology, cholecystectomy has been further widely applied worldwide, and more and more patients are undergoing or have undergone cholecystectomy (Harrison et al., 2012; Tiderington et al., 2016). However, in addition to serious surgery-related complications (such as bile duct injury, bleeding, bile leakage, and infection), surgical removal of the functional gallbladder in patients with gallstones may also lead to some physiological changes in the gastrointestinal tract (Sicklick et al., 2005). Cholecystectomy changes the way bile drains into the intestinal cavity and increases the enterohepatic recirculation rate of bile acids. It also increases the exposure of intestinal flora to bile acid pools, eventually leading to an increase in the proportion of secondary bile acids (SBAs) (Wang et al., 2018). Postcholecystectomy may affect the long-term prognosis of patients. In addition to an increased risk of colorectal cancer (Goldacre et al., 2012), cholecystectomy is also associated with an increased risk of other gastrointestinal cancers and metabolic syndromes (such as insulin resistance, weight gain, dyslipidemia, hepatic steatosis, and hypertension) (Saklayen, 2018; Uldall Torp et al., 2020).

Colorectal cancer (CRC) is a relatively common cancer with a high degree of malignancy. Although its incidence is lower than lung cancer and stomach cancer, its mortality rate ranks second place. More and more clinical data show an increasing incidence in young and middle-aged people accompanied by a higher degree of malignancy (Saraste et al., 2020). The global estimated crude incidence of colon cancer in 2020 was 14.7 per 100,000, and the standardized incidence was 11.4 per 100,000. The crude mortality rate was 7.4 per 100,000, and the standard mortality rate was 5.4 per 100,000. The global estimated crude incidence of rectal cancer was 9.4 per 100,000, and the standard incidence was 7.6 per 100,000. The crude mortality rate was 4.3 per 100,000, and the standard mortality rate was 3.3 per 100,000. In 2020, the estimated incidence of colon cancer in our country was 26.6% of the world, with a crude incidence of 21.1/100,000 and a standard rate of 13.1/100,000. Colon cancer is estimated to account for 28.6% of global deaths, with a crude mortality rate of 11.4 per 100,000 and a standardized rate of 6.8 per 100,000. It is estimated that the incidence of rectal cancer accounts for 33.4% of the global population, with a crude incidence of 16.9 per 100,000 and a standardized rate of 10.6 per 100,000. Colorectal cancer is estimated to account for 35.1% of global deaths, with a crude mortality rate of 8.2 per 100,000 and a standardized rate of 5.0 per 100,000 (Sung et al., 2021).

Colorectal cancer is a complex disease, the cause and mechanism of which have not been fully elucidated so far. Known influencing factors include age, genetic factors, diet, metabolic diseases, and environment (Cho et al., 2019; De Almeida et al., 2019; Song and Chan, 2019). With the rise of molecular biology in recent years, through the application of gene sequencing technology, metabolomics, and other applications, more and more studies have found that intestinal flora disorders are closely related to the occurrence and development of CRC (Bundgaard-Nielsen et al., 2019). In recent years, several scholars have studied the intestinal flora after cholecystectomy, and the research consensus is that cholecystectomy changes the intestinal flora composition of patients and puts them at a high risk of colorectal cancer (Chen et al., 2014, 2020; Ábrahám et al., 2020).

At present, many scholars have conducted in-depth studies on the relationship and its mechanism between cholecystectomy and colorectal cancer through case–control studies, prospective and retrospective cohort studies, animal experimental studies, etc. Most scholars believe that cholecystectomy can increase the risk of colorectal cancer. However, its relationship with colorectal cancer after cholecystectomy and its mechanism remain unclear. In view of this, this article will combine the research progress at home and abroad in recent years, with the theme of “Relationship between Cholecystectomy and Colorectal Cancer” and make a review.

## 2. Relationship between cholecystectomy and colorectal cancer

### 2.1. Epidemiological relationship between cholecystectomy and colorectal cancer

Most domestic and foreign studies suggest that cholecystectomy can increase the risk of colorectal cancer. A meta-analysis covering 33 case–control studies showed an association between cholecystectomy and risk of colorectal cancer (pooled relative risk [RR] = 1.34; 95% confidence interval [CI] = 1.14–1.57), particularly when limited to the proximal colon (RR = 1.88; 95% CI = 1.54–2.30) (Giovannucci et al., 1993). Another meta-analysis of nine cohort studies showed that an increased risk of CRC was found among the individuals who had undergone cholecystectomy [risk ratio (RR) 1.22; 95% confidence interval (CI) 1.08–1.38]. In addition, we also found a promising increased risk for colon cancer (CC) (RR 1.30, 95% CI 1.07–1.58), but no relationship between cholecystectomy and rectum cancer (RC) (RR 1.09; 95% CI 0.89–1.34) was observed. Furthermore, a positive relationship between the female gender and CRC was also found (RR 1.17; 95% CI 1.03–1.34) (Zhang et al., 2017a). Xu et al. (2009) and Chiong et al. (2012) confirmed an increased incidence of colorectal cancer in individuals who underwent cholecystectomy. Kim et al. (2020) studied the association between cholecystectomy and stomach or colorectal cancer in Korean individuals by collecting data about patients who underwent cholecystectomy at their institutions over an 8-year period (excluding patients with other gastrointestinal cancers and those diagnosed with stomach or colorectal cancer within 1 year of cholecystectomy). They found that the risk of CRC increased up to 108% after cholecystectomy in the patients compared with that in the general population with statistical significance. Moreover, the risk of CRC increased in female patients compared with that in the general population when analyzed by sex. The male and female participants had a 74 and 154% higher risk of CRC, respectively. In recent years, relevant literature has been updated continuously, but the analysis results still maintain a high consistency. These results suggest that the history of cholecystectomy is closely related to the occurrence and development of colorectal cancer.

There are also scholars who hold opposing views on this issue. A retrospective study in Hungary found that although a statistically higher risk of colon cancer was observed after gallbladder removal, the incubation period was long. Therefore, cholecystectomy may not be an independent risk factor for colorectal cancer (Mándi et al., 2021). A Japanese study revealed a negative association between cholecystectomy and colorectal cancer (Kaibara et al., 1986). Other studies have shown no relationship between cholecystectomy and colorectal cancer (Vinikoor et al., 2007; Zhao et al., 2012).

### 2.2. Effect of cholecystectomy on the anatomical location of colorectal cancer

Most studies suggest that cholecystectomy is closely related to the occurrence of right colon cancer, but there is no obvious correlation with rectal cancer. Shao and Yang (2005) evaluated the relationship between cholecystectomy and colorectal cancer and found that cholecystectomy could promote the occurrence of colon cancer, especially right-sided colorectal cancer, and had nothing to do with the occurrence of rectal cancer and left-sided colorectal cancer. A small number of studies believe that cholecystectomy also has a certain impact on the occurrence of rectal cancer. Some studies have found that the increased risk of proximal colon cancer and rectal cancer after cholecystectomy is statistically significant (Schernhammer et al., 2003). Giovannucci et al. (1993) proposed that after cholecystectomy, CRC was more likely to occur in the proximal colon, which might be due to the difference in the origin of the proximal and distal colon embryos, so there was a difference in susceptibility to tumor formation.

### 2.3. The interval of colorectal cancer after cholecystectom

Some scholars believe that the effect of cholecystectomy on colorectal cancer is a unimodal wave, and the risk of colorectal cancer after the operation gradually increases and gradually decreases after reaching the peak (Zhang et al., 2021). Some studies found that cholecystectomy has a positive effect on the occurrence of colorectal cancer, but the positive effect disappears after 10 years (Altieri et al., 2004). Chen et al. (2014) followed up patients after cholecystectomy and found that the incidence of colorectal cancer increased within 5 years after cholecystectomy, but no significant effect was observed 5 years later. Lee et al. (2018) also reached a consistent conclusion through their findings; a higher risk of colorectal cancer was observed 1–5 years after cholecystectomy.

## 3. Analysis of the relationship between cholecystectomy and colorectal cancer from the pathogenesis

### 3.1. Changes in bile acid metabolism after cholecystectomy cause colorectal cancer

In recent years, studies have shown that significant changes in bile acid metabolism after cholecystectomy may be the main cause of colorectal cancer induction, but the specific mechanism of colorectal cancer induction after cholecystectomy remains unclear. According to the research progress at home and abroad, the changes in bile acid metabolism after cholecystectomy and the mechanism of inducing colorectal cancer were briefly reviewed.

#### 3.1.1. Changes of bile acid metabolism after cholecystectomy

Studies have shown that the bile acid pool of patients undergoing cholecystectomy decreased 3 months after surgery, and the total amount of bile acids decreased by approximately 16%, while the total amount of bile acids remained basically unchanged 5~8 years after surgery (Kullak-Ublick et al., 1995). Another study found that the synthesis rate of anoxycholic acid and the capacity of the bile acid pool decreased 6 weeks after cholecystectomy, but the synthesis rate of anoxycholic acid and deoxycholic acid and the size of the bile acid pool did not change significantly 9 to 12 months after surgery (Zhao et al., 2022). These results suggest that the synthesis of bile acids is inhibited after cholecystectomy, and the size of the bile acid pool is reduced in the short term, but there is no significant effect in the long term.

After cholecystectomy, bile loses its storage place, and the internal pressure in the biliary tract is higher than the pressure of the Oddi sphincter, which leads to the long-term opening of the Oddi sphincter, resulting in the continuous discharge of bile produced by the liver into the intestine and an increase of the enterohepatic circulation of bile acids (Han et al., 2012). The amount of enterohepatic circulation of bile acids after cholecystectomy is twice that of healthy people (Sergeev et al., 2020). Clinical studies have shown that after cholecystectomy, the hepatoenteric circulation of bile acids will be accelerated, the content of cholic acid (CA) in intestinal bile acid will be decreased, the content of deoxycholic acid (DCA) and lithocholic acid (LCA) will be increased (Roda et al., 1978), and the content of SBAs in fecal bile acids will be increased (Zuccato et al., 1993). Studies have found that after cholecystectomy, bile acid circulation increases, the contact between primary bile acid and intestinal flora increases, the dehydroxylation of primary bile acid is enhanced, and the concentration of SBAs (especially stone cholic acid) in bile is significantly increased (Zhai et al., 2019). In addition, cholecystectomy increases bacterial decoupling and dehydroxylation of bile acids, thereby increasing the proportion of SBAs (Housset et al., 2016). Zhang et al. (2017b) results showed that deoxycholic acid, lithocholic acid, and their combined products with taurine significantly increased in ileal contents of mice after cholecystectomy, and fecal bile acids also increased accordingly.

#### 3.1.2. Bile acid-induced mechanism of colorectal cancer occurrence

##### 3.1.2.1. Dysbiosis of gut microbiota

Studies have shown that there is an interaction between bile acid metabolism and intestinal microflora (Chunyang et al., 2019). After cholecystectomy, bile synthesis, secretion, concentration, excretion, and reabsorption are abnormal. A low concentration of bile acids reduces the antibacterial effect, which may cause excessive proliferation of intestinal bacteria. A low concentration of bile acids can reduce the transepithelial resistance of the colon tissue, increase the permeability of the intestinal epithelium, and increase the absorption of bacteria in the colon tissue. The reduced reabsorption of bile acids in the small intestine leads to the increase of bile acids discharged into the colon, which promotes the increase of the production of SBAs, enhances the inhibition of beneficial bacteria, and further leads to intestinal flora disorder.

##### 3.1.2.2. Secondary bile acids are an important link in the development of colorectal cancer

High levels of SBAs and deoxycholic acid (DCA) in fecal matter and patient's serum correlate with increased adenomatous polyp recurrence and the development of higher grade, and more aggressive disease (Bayerdörffer et al., 1993). Animal experiments show that bile acids, especially DCA and LCA, can enhance the effect of cancer inducers on colorectal cancer (Zusman and Zimber, 1991). Bo Yang et al. conducted a study on the effect of cholecystectomy on colorectal tumors in mice caused by dimethylhydrazine (DMH) and found through a mice colon cancer induction experiment that cholecystectomy could increase the S-phase cells of the colon mucosal epithelium in mice, indicating that cholecystectomy can promote the occurrence of colon cancer, which may be related to the increased content of lithocholic acid in SBAs in the intestinal tract after operation (Bo et al., 2002). Kuniyasu et al. (1986) induced large intestinal carcinogenesis in hamsters using methylazoxymethanol (MAM), and the results indicate an enhancing effect of cholecystectomy on MAM acetate-induced large intestinal carcinogenesis in hamsters, which may be related to abnormal metabolism of secondary bile acid in the intestinal tract after operation.

At the cellular level, SBAs promote the proliferation of normal colorectal cells and tumor cells at the same time by changing the morphology and dynamics of colonic mucosa cells (Ajouz et al., 2014). Bile acids, including SBAs, are hydrophobic and therefore may alter the stability of the membrane lipid bilayer, and have the capacity to disturb the structure of, or partly digest, cell membranes (Sagawa et al., 1993). Secondary bile acids (DCA and LCA) also increased paracellular permeability in a dose-related manner, with LCA exerting more potent effects than DCA (Stenman et al., 2012). When present in high concentrations, SBAs cause unspecific cell membrane damage, resulting in focal destruction of intestinal epithelium (Payne et al., 2008). SBAs reduce immune function by inhibiting the proliferation of intestinal mucosa lamina propria lymphocytes (Collins et al., 2023).

At the molecular level, secondary bile acids can cause DNA damage, directly interfere with DNA metabolism, increase thymidine insertion and DNA synthesis outside normal sequence, improve ornithine decarboxylase activity in colonic mucosa cells, and induce multi-gene mutations of oncogenes, onco-suppressor genes, and mismatch repair genes (Ajouz et al., 2014; Niekamp and Kim, 2023). Secondary bile acids can induce genomic instability in colonic epithelial cells through multiple mechanisms, including the disruption of mitosis (leading to aneuploidy), defects in spindle assembly checkpoints, oxidative DNA damage, cell cycle arrest at G1 and/or G2 along with an improper alignment of chromosomes at the metaphase plate and multipolar divisions (Degirolamo et al., 2011). DCA stimulates extracellular signal-regulated kinases, which counteracts the tumor-suppressive activity of the tumor suppressor p53 (Qiao et al., 2001a). DCA impaired p53 signaling in a cancer cell line exposed to DNA-damaging reagents *in vitro* (Qiao et al., 2001b).

##### 3.1.2.3. Secondary bile acids influence molecular signaling of colorectal cancer pathogenesis

At present, it has become clear that bile acids can also act as versatile signaling molecules and even regulate gene expression. Bile acids have been discovered to activate specific nuclear receptors (farnesoid X receptor, preganane X receptor, and vitamin D receptor), G-protein-coupled receptor TGR5 (TGR5), and cell signaling pathways (c-jun N-terminal kinase 1/2, AKT, and ERK 1/2) in cells in the liver and gastrointestinal tract. Activation of nuclear receptors and cell signaling pathways alter the expression of numerous genes encoding enzymes/proteins involved in the regulation of bile acid, glucose, fatty acid, lipoprotein synthesis, metabolism, transport, and energy metabolism (Hylemon et al., 2009). Major pathways implicated in colon cancer development include WNT signaling, phosphatidylinositol 3-kinase (PI3K) signaling, MAPK signaling, p53 deregulation, and more recently prostaglandin E2 (PGE2) signaling (Centuori and Martinez, 2014). Secondary bile acids increase the risk of colorectal cancer through the *epidermal growth factor receptor (EGFR), Wnt/*β*-catenin*, and *protein kinase C* signaling pathways (Bingfeng et al., 2020). Yao (2018) identified the key role of secondary bile acids (especially deoxycholic acids) in the occurrence and development of colorectal cancer, namely, they mainly trigger colorectal cancer through the four pathways of *NF Kb, Wnt/*β*-catenin, mitogen-activated protein kinase (MAPK)*, and *miRNA*.

Sustained exposure to deoxycholic acid (DCA) in the colon epithelium after cholecystectomy may have cytotoxic effects that promote colon cancer progression by inhibiting FXR expression and enhancing the Wnt-β-catenin pathway (Yao et al., 2022). The canonical WNT pathway is activated when a number of WNT ligands bind to Frizzled receptors. This interaction activates disheveled (DSH), which, then, inhibits the complex of proteins composed of axin, GSK-3β, and APC. This complex usually promotes the proteolytic degradation of β-catenin. By activating WNT signaling, this degradation is blocked, and β-catenin is able to accumulate in the cytoplasm and then translocate to the nucleus where it is able to interact with TCF and LEF transcription factors, resulting in upregulation of specific genes involved in cellular proliferation (Logan and Nusse, 2004).

β-Catenin is a key component of adherens junctions that link the actin cytoskeleton to members of the cadherin family of transmembrane cell–cell adhesion receptors. Tyrosine phosphorylation of β-catenin has been suggested to promote metastatic potential and tumor invasiveness by stabilizing β-catenin and promoting its binding of T cell factor (TCF)/lymphocyte enhancer factor 1 DNA transcription factors. The latter complex functions as a transcriptional activator and plays a key role in regulating cancer cell proliferation and metastasis. Transcriptional targets of β-catenin/TCF include cyclin D1, urokinase plasminogen activator receptor (uPAR), matrix metalloproteinase 7, cyclooxygenase-2, gastrin, and CD44 (Wong and Pignatelli, 2002). The experiment proved that physiologically relevant concentrations (5 or 50 μM) of DCA activate β-catenin signaling pathway and increase urokinase plasminogen activator (uPA), uPAR, and cyclin D1 expression in colon cancer cells. Inhibition of β-catenin expression by using siRNA targeted for β-catenin significantly suppressed DCA-induced uPAR and cyclin D1 expression. On the basis of these data, they proposed the following sequence of events. Deoxycholic acid-induced tyrosine phosphorylation stabilizes and translocates β-catenin into the nucleus and stimulates uPA, uPAR, and cyclin D1 expression. Tyrosine phosphorylation dissociates β-catenin from E-cadherin and thus induces loss of cell adhesion. uPA/uPAR–mediated proteolytic degradation of extracellular matrix accompanied by cyclin D1/uPA/uPAR-induced cell proliferation enhances colon cancer growth and progression (Pai et al., 2004).

Previous studies have shown that low concentrations of DCA (< 50 μM) can increase the proliferation of colon cancer cells, while high concentrations of DCA (>100 μM) can increase apoptosis (Peiffer et al., 1997). Colon cancer cells treated with higher concentrations of DCA are sufficient to induce strong activation of EGFR-MAPK signaling (Qiao et al., 2000). Activation of the EGFR signaling pathway by secondary bile acids is achieved mainly by disturbing the structure of the cell membrane (reduced membrane fluidity, altered membrane cholesterol distribution), binding to natural ligands (e.g., epidermal growth factor), or inducing calcium signaling-mediated non-dependent activation of ligands. Activation of EGFR activates downstream MAPK/RAS/RAF/MEX/extracellular signal-regulated kinase/proto-oncogene activator protein-1, which, in turn, mediates cell proliferation and activates RAS/RAF1/extracellular signal-regulated kinase signaling pathway, leading to upregulation of mucin 2 and also activates the phosphatidylinositol 3 kinase/Akt signaling pathway, which regulates downstream target molecules such as caspase-8, leading to apoptosis (Centuori et al., 2016).

Another molecule with tumor-promoting activities in colon cells is cyclooxygenase 2 (COX-2). DCA leads to COX-2 activation and increased expression. Stimulation of COX-2 leads to increased PGE2 levels, another molecule whose expression is enhanced by the presence of DCA. Once PGE2 production is induced in the colon cells, it is able feedback in an autocrine fashion that stimulates a complex signaling cascade. This stimulation of signaling leads to increased cell survival, proliferation, angiogenesis, and resistance to apoptosis (Khare et al., 2008).

DCA has also been documented to decrease the levels of p53, a tumor suppressor that regulates cell cycle progression. The loss of p53 is an integral requirement for the development of many tumors including adenocarcinomas of the colon. A study has investigated the effect of bile acids on the tumor suppressor p53 using the human colon tumor cell line HCT116. They found that exposure of the cells to elevated concentrations of DCA suppressed the accumulation of p53 protein as well as p53 transactivation and impaired the p53 response of the cells to DNA-damaging agents, such as ionizing radiation. The study suggested that DCA suppressed p53 by stimulating the process of proteasome-mediated degradation of p53, in part, by stimulating the ERK signaling pathway (Qiao et al., 2001b).

### 3.2. Dysbiosis of intestinal flora after cholecystectomy causes colorectal cancer

Intestinal flora is involved in physiological, biochemical, and pathological processes of the body. It has the function of promoting immune regulation and intestinal nervous system development, preventing pathogen invasion, maintaining normal intestinal movement, and inhibiting tumor occurrence and development. Intestinal flora changes after cholecystectomy, but the specific mechanism is still unclear and needs to be explored through a large number of basic and clinical experimental studies. Based on the research at home and abroad, this study summarizes the current research in order to provide a new target for the treatment of gallbladder diseases.

#### 3.2.1. Changes of intestinal flora after cholecystectomy

The study showed that the gut microbiome of patients undergoing cholecystectomy is significantly different from that of healthy individuals but similar to that of patients with colorectal cancer, suggesting that changes in the gut microbiome of patients undergoing cholecystectomy may activate the onset and progression of colorectal cancer (Ren et al., 2020). Cholecystectomy causes dramatic changes in the intestinal microecology, including the composition and function of the intestinal microbiome. Previous studies confirmed that after cholecystectomy, the number of *Bifidobacteria* and *Lactobacillus* were significantly decreased, while the number of *Enterococcus, Oscillospira, Escherichia coli, Bacteroidaceae*, and *Bacteroidetes* were significantly increased (Jiang et al., 2022). At the phylum level, the abundance of *Fusobacteria* increased, whereas that of *Proteobacteria* decreased. Other phyla, including *Bacteroidetes, Firmicutes*, and *Actinobacteria* showed distinct variations in different studies. Interestingly, the changes in bacterial abundance of *Firmicutes* and *Actinobacteria* were similar in all studies, in contrast to the alteration in the bacterial abundance of *Bacteroidetes*. At the genus level, existing research has not reached a consensus. Genera reported with increasing abundance mainly include *Anaerostipes, Dorea, Clostridium, Mogibacterium, Flavonifractor, Shigella*, and *Escherichia*, while those with reduced abundance include *Paraprevotella, Prevotella, Barnesiella, Alistipes, Faecalibacterium, Haemophilus*, and *Desulfovibrio*. Few studies have focused on the species level; *Blautia obeum, Veillonella parvula, Bacteroides ovatus, Parabacteroides distasonis*, and *Fusobacterium varium* were found to increase, and *Eubacterium rectale, Roseburia faecis*, and *Bifidobacterium adolescentis* were reported to decrease (Ma et al., 2022). Ren et al. (2020) described the overall structure of bacterial microbiota by 16S rRNA gene sequencing, proving that bacterial ecological disorders after cholecystectomy are characterized by unique microbial composition and changes in the relative abundance of species with specific functions. Wang et al. (2012) and Wu et al. (2013) conducted a comparative analysis of fecal flora in colorectal cancer patients and healthy people in China using 16SrRNA pyrosequencing. The results showed that colorectal patients have a higher number of pathogenic bacteria compared with healthy people, especially *Enterococcus, Shigella, Klebsiella, Streptococcus*, and *Streptococcus*. In addition, evidence from foreign studies shows that the fecal abundance of *Clostridium, Porphyrophyria*, and *Strangella* in patients with colorectal cancer increases (Ahn et al., 2013). At present, most domestic scholars believe that intestinal *Bifidobacterium* and *Lactobacillus* are significantly reduced after cholecystectomy, while *Escherichia coli* and *Enterococcus* are significantly increased (Hongguang and Deqing, 2015).

#### 3.2.2. Mechanism of intestinal flora dysregulation after cholecystectomy

##### 3.2.2.1. Imbalance of bile acid metabolism

Fibroblast growth factor (FGF) is a cytokine synthesized by terminal ileal epithelial cells, which is involved in regulating bile acid metabolism. FGF19 or FGF15 produced in the ileum is transported to the liver via the portal system to inhibit bile acid synthesis. It has been found that the mRNA content of FGF19 in gallbladder epithelial tissue is 250 times more in terminal ileal epithelial tissue. After cholecystectomy, with the decrease of FGF19 expression, the metabolic balance of bile acids is broken, and the production of primary bile acid increases, which changes the two-way interaction between bile acid and intestinal flora (Barrera et al., 2015; Keren et al., 2015).

Elevated deoxycholic acid (DCA) in the gut inhibits the growth of most *Gram-negative anaerobic bacteria*, such as *Bacillus fragilis, Clostridium perfringens, Lactobacillus, Bifidobacterium*, and *Enterococcus* (Floch et al., 1972). In addition, Cao et al. (2017) found that DCA significantly upregulated the populations of opportunistic pathogens, including *Ruminococcus, Escherichia-Shigella, Desulfovibrio*, and *Dorea*. Moreover, they also confirmed that DCA significantly increased the levels of *Clostridium* and *Escherichia-Shigella* but markedly decreased the abundance of *Lactobacillus_gasseri* and mostly butyrate-producing bacteria, such as *Clostridium leptum Lachnospiraceae bacterium*, and *Eubacterium coprostanoligenes* (Cao et al., 2017).

##### 3.2.2.2. Immune abnormality

The mucosal epithelium of the gallbladder synthesizes a surfactant protein D, which is discharged into the intestinal cavity along with the bile to promote the synthesis of intestinal T cells. Intestinal T cells are involved in regulating inflammation in the gut. After cholecystectomy, the number of intestinal T cells decreases sharply due to the lack of gallbladder surface protein D, which is easy to cause intestinal bacterial infection, resulting in intestinal flora imbalance. Gallbladder surface protein D can also inhibit the growth of intestinal *Lactobacillus* by directly binding to *Lactobacillus* and causing its cleavage (Boehm et al., 2012; Sarashina-Kida et al., 2017).

##### 3.2.2.3. Acid–base imbalance

The small intestine fluid is weakly alkaline with a pH of 8.0~9.0. Normal bile is weakly acidic, and its impulsive secretion is conducive to shaping a good intestinal microecological environment and maintaining the stability of intestinal pH value. After cholecystectomy, alkaline hepatic bile secrets continuously, which affects the balance of pH in the intestine. The optimum pH values of *Lactobacillus* and *Bifidobacterium* are 5.5~6.0 and 6.5~7.0, respectively. Therefore, the rise of pH in the intestine inhibits the growth of *Lactobacillus, Bifidobacterium*, and other beneficial bacteria, resulting in the imbalance of intestinal flora (Lin et al., 2018).

#### 3.2.3. The carcinogenic mechanism of intestinal flora changes after cholecystectomy

##### 3.2.3.1. Inflammation and immune regulation

Intestinal microbiota has the potential to form an inflammatory microenvironment, and inflammation is a well-established risk factor for colorectal cancer. A study showed that specific components of the gut microbiome promote the development and progression of colorectal cancer through the activation of inflammation (Sears et al., 2014).

Intestinal homeostasis is achieved through an ongoing interaction between the gut microbiome and the host immune system. Once this balance is upset, a variety of diseases, such as inflammatory bowel disease, can arise as a result of immune system dysfunction (Gophna et al., 2006).

##### 3.2.3.2. The production of genotoxins

Another oncogenic mechanism of the gut microbiome is the production of genotoxins, which can cascade with intracellular signals or cause mutations by binding to specific cell surface receptors, which can also damage DNA (Valguarnera and Wardenburg, 2020). Interestingly, although many genotoxins can cause tumors, recent research has shown their potential use in cancer treatment (Zahaf and Schmidt, 2017).

##### 3.2.3.3. Metabolism of dietary components

Bacteria in the gut produce a range of metabolites, including secondary bile acids, sulfides, ammonia, nitrosamines, and short-chain fatty acids (SCFAs), which are involved in the development and progression of colorectal cancer. Dietary fiber is metabolized and broken down into SCFAs in the colon, of which butyrate (the most widely studied SCFA) regulates cell proliferation, apoptosis, and differentiation to inhibit colorectal tumors. Cholecystectomy greatly reduces the number of gut bacteria responsible for metabolizing butyrate. Therefore, the expression level of butyrate is decreased, and the occurrence of colorectal tumors is promoted (Ren et al., 2020). Gut microbiota can also disrupt mucus barrier function by producing sulfides, thus enhancing intestinal cell stimulation (Ijssennagger et al., 2015).

##### 3.2.3.4. Activation of signaling pathways

Multiple signaling pathways, such as *epithelial growth factor receptor* (*EGFR*), *Wnt/beta-catenin, NF-*κ*B*, and *transforming growth factor-*β pathways, are involved in the development and progression of colorectal cancer. Notably, the gut microbiome activates the host carcinogenic signaling pathway (Ma et al., 2022).

It was reported that Enterotoxigenic *Bacteroides fragilis(ETBF)* secrete a zinc-dependent metalloprotease toxin termed the *B. fragilis toxin (BFT)*. *BFT* rapidly cleaves the extracellular domain of *E-cadherin*, leading to the complete degradation of the *E-cadherin* protein, triggering β-catenin nuclear signaling in intestinal epithelial cells, and inducing *c-Myc* expression and cellular proliferation (Wu et al., 2003). Chung et al. constructed ApcMin mice colonized with an enterotoxigenic *B. fragilis* strain, possessing an in-frame chromosomal deletion of the *bft* gene. The results showed that *B. fragilis* stimulated intracellular *IL-17* secretion to activate the *NF-*κ*B* pathway, which, in turn, induced the expression of chemokines *(CXCL1, CXCL2*, and *CXCL5)* that collectively contributed to colonic carcinogenesis (Chung et al., 2018). The cytokine *IL-8*, a key downstream target gene of *NF-*κ*B*, was also significantly increased in intestinal epithelial cells treated with active enterotoxigenic *B. fragilis* (Wu et al., 2004). The β*-catenin* and *GSK3*β cellular signaling pathways are involved in *NF-*κ*B* activity and *IL-8* expression in *B. fragilis*-infected cells (Jeon et al., 2019).

A growing body of evidence suggests a potential link between the *Fusobacterium nucleatum (F. nucleatum)* and colorectal carcinogenesis. *Fusobacterium nucleatum* is enriched in human colorectal adenomas and carcinomas compared with adjacent normal tissue (Kostic et al., 2013; Ito et al., 2015). A marked accumulation of *Fusobacterium nucleatum*, which expresses *FadA* adhesin on its surface, was found in patients with CRC. *Fusobacterium nucleatum* adheres to and invades the endothelial and epithelial cells through its unique *FadA* adhesin. *FadA* modulates *E-cadherin* and activates β*-catenin* signaling, leading to increased expression of transcription factors, oncogenes, *Wnt* genes, and inflammatory genes, as well as growth stimulation of CRC cells (Rubinstein et al., 2013). *Fusobacterium nucleatum* can also directly activate Toll-like receptor signaling to promote tumor development (To et al., 2015). It has been shown that *F. nucleatum-*mediated infection leads to a strong induction of *TLR4/MYD88* and results in the sustained activation of *NF*κ*B*. The hyperactive *NF*κ*B* binds to the promoter of *miR21* and increases its transcription level. *MiR21* is a downstream target of *F. nucleatum*, and *F. nucleatum* regulates the expression of *miR21* novel target *RASA1* and activates the *MAPK* signaling pathway, consequently leading to the initiation of CRC (Yang et al., 2017).

The *NF-*κ*B* family of transcriptional factors regulates a large number of genes involved in different cellular processes, such as cell proliferation, differentiation, genome stability, and innate immune and adaptive immune responses (Liu et al., 2017). The *NF-*κ*B* signaling pathway can be activated to modulate host cellular events after exposure to different microbial pathogens or microbial products, such as lipopolysaccharide (LPS) and pathogen-associated molecular patterns (PAMPs) (Rahman and McFadden, 2011). Cytoplasmic *NF-*κ*B* is transferred to the nucleus, where it induces antimicrobial inflammatory cytokine expression, which functions as a rapid defense mechanism against microbes, including infectious bacteria. However, prolonged chronic inflammation due to the activation of *NF-*κ*B* proteins may result in tissue damage, further contributing to tumorigenesis by changing the genetic and epigenetic states of damaged tissues and the host microenvironment (Taniguchi and Karin, 2018). *Fusobacterium nucleatum (F. nucleatum), Escherichia coli (E. coli), Peptostreptococcus anaerobius (P. anaerobius)*, and Enterotoxigenic *Bacteroides fragilis (ETBF)*, which are enriched in patients with CRC, are involved in the modulation of this pathway (Peng et al., 2020). Enteropathogenic and enterohemorrhagic *E. coli* use a type III secretion system (T3SS) to transport dozens of effector proteins into host cells; these effector proteins, in turn, manipulate the host inflammatory response through activation of the *NF-*κ*B* pathway (Litvak et al., 2017). Mechanistically, internalization of *E. coli* leads to the activation of *NF-*κ*B* through increased phosphorylation of the *NF-*κ*B* subunit *RelA/p65* and *IKK*α, inactivation of *I*κ*B*α, and induction of the *Wnt/*β*-caten*in pathway through the upregulation of β*-catenin* and its downstream genes. Then, *NF-*κ*B* and *Wnt/*β*-catenin* synergistically promote tumorigenic stemness traits (Sahu et al., 2017). *P. anaerobius* interacts with *TLR2* and *TLR4* on colon cells to increase the levels of reactive oxidative species, which promotes cholesterol synthesis and cell proliferation (Tsoi et al., 2017). *P. anaerobius* adheres to the CRC mucosa and accelerates CRC development. A study identified a *P. anaerobius* surface protein, *putative cell wall binding repeat 2 (PCWBR2)*, which directly interacts with colonic cell lines via α2/β1 integrin. Interaction between *PCWBR2* and integrin α*2/*β*1* induces the activation of the *PI3K-Akt* pathway in CRC cells via phospho-focal adhesion kinase, leading to increased cell proliferation and *nuclear factor kappa-light-chain-enhancer of activated B cells (NF-*κ*B)* activation. *NF-*κ*B*, in turn, triggers a pro-inflammatory response as indicated by increased levels of cytokines (Long et al., 2019).

### 3.3. Gene mutation theory

Several specific and cumulative genetic changes occur during colorectal tumorigenesis, including mutation and activation of the cellular proto-oncogene *Ki-ras* and inactivation of tumor suppressor genes such as *P53, hMSH2, APC*, and *DCC*. If the above genetic alterations occur in colorectal epithelial cells (EC) in patients with cholelithiasis, the correlation between cholelithiasis and CRC can be evaluated in comparison with the healthy population without cholelithiasis (Werner et al., 1977).

The expression of *Ki-67* in intestinal mucosa after cholecystectomy was significantly higher than that in non-cholecystectomy, indicating that intestinal mucosa was in a state of high proliferation after cholecystectomy (Jager et al., 2016).

The study showed that the probability of developing dMMR colorectal cancer after cholecystectomy was not significantly different from that of non-dMMR colorectal cancer. Therefore, the theory that cholecystectomy causes DNA mismatch repair dysfunction in colorectal cells still needs to be further confirmed by more studies (Skagen et al., 2009).

### 3.4. Humoral factor theory

Gastrin increased significantly in blood circulation after cholecystectomy. Gastrin is a trophic growth factor of colon epithelial cells, which can activate a variety of signaling pathways, such as the *HedgeHog* signaling pathway, the *Wnt* signaling pathway, and the *MAPK* signaling pathway, and promote the occurrence and development of colorectal cancer (Redaelli et al., 2002; Wang et al., 2011).

Studies have shown that the concentration of cholecystokinin increases after cholecystectomy, which can be used as a ligand to activate the cholecystokinin II receptor (*CCK2R*). The upregulation of *CCK2R* can cause the transformation of human non-tumorigenic *NCM356* colon epithelial cells into tumor-germinating cells (Chao et al., 2010).

### 3.5. Gallstone itself is a risk factor for colorectal cancer

Since gallstones are the main reason for cholecystectomy, some scholars believe that gallstones are a risk factor for colorectal cancer. A study which to systematically review and meta-analyze the association between the presence of gallstone disease (GD) or cholecystectomy (CE) and the incidence of colorectal cancer (CRC) showed that the overall association of GD and/or CE with CRC was RR = 1.15 (1.08; 1.24), primarily driven by hospital-based case-control studies [RR = 1.61 (1.29; 2.01)]. whereas a more modest association was found in population-based case-control and cohort studies [RR = 1.10 (1.02; 1.19)]. This study showed that gallstones were associated with a moderately increased risk of colon cancer (Polychronidis et al., 2023). Pinlian et al. (2012) believed that gallstones had adverse effects on the intestinal microenvironment and even led to precancerous lesions before cholecystectomy. Gallstones can cause inflammation of the gallbladder, bile ducts, liver, and pancreas, so the link between inflammation and cancer is well established, and as a result, gallstones can increase the risk of many different digestive cancers (Gosavi et al., 2017). Some scholars believe that the factors that can promote the formation of gallstones may also be the inducement of colorectal cancer, such as genetic predisposition, female sex, obesity, high fat, and low fiber diet (Völzke et al., 2005).

Recently, several studies have attempted to explore the causal relationship between cholecystectomy and colorectal cancer (CRC) from the genetic perspective. Studies have confirmed that gallstone disease is associated with an increased risk of CRC (Chen et al., 2023; Tsai et al., 2023). Chen et al. (2023) obtained genetic variants associated with cholecystectomy at a genome-wide significant level (*P*-value < 5 × 10–8) as instrumental variables (IVs) and performed Mendelian randomization (MR) to identify the complications of cholecystectomy. Furthermore, cholelithiasis was also treated as the exposure to compare its causal effects with that of cholecystectomy, and multivariable MR analysis was carried out to judge whether the effect of cholecystectomy was independent of cholelithiasis. They found that cholelithiasis could increase the risk of CRC in the largest population (OR = 1.041, 95% CI: 1.010–1.073). The multivariable MR analysis suggested that genetic liability to cholelithiasis could increase the risk of CRC in the largest population (OR = 1.061, 95% CI: 1.002–1.125), after adjustment of cholecystectomy. Another study came to a consistent conclusion, showing that cholelithiasis is associated with an increased risk of CRC, especially in the right-sided colon and among female patients. Cholecystitis and cholecystectomy may shift cancer to the distal part of the large bowel (Tsai et al., 2023).

However, research has found that increased production of bilirubin, the metabolic by-product of hemoglobin degradation, is also associated with gallstone disease (Kühn et al., 2017). This is intriguing as bilirubin has antioxidant and anti-inflammatory attributes, with experimental studies reporting mildly elevated levels being associated with decreased oxidative stress-related disease, including cancer (Vítek, 2012).

At present, the cholecystolithotomy technique of natural orifice transluminal endoscopic surgery is evolving, with some experts advocating gallbladder stone removal without gallbladder excision in order to preserve gallbladder function and eliminate post-cholecystectomy syndromes, including complications of the surgical incision, bile duct injury, functional gastrointestinal, and psychological conditions, and possibly an increase in colon cancer. In addition, transluminal endoscopic cholecystolithotomy is an option for elderly patients who are not suitable candidates for open surgery and those who desire scar-free minimally invasive surgery with organ preservation (Shang et al., 2022).

## 4. Conclusion

In summary, epidemiological studies on colorectal cancer and cholecystectomy have demonstrated the correlation between the two parameters, and most scholars support that cholecystectomy increases the risk of colorectal cancer. Therefore, patients after cholecystectomy should be followed up regularly to detect colorectal tumors early. In addition, since cholecystectomy may induce colorectal cancer, the pros and cons should be weighed when performing cholecystectomy, to reduce unnecessary cholecystectomy. The authors believe that with continuous research on the mechanism of the occurrence and development of colorectal cancer induced by cholecystectomy, new methods for targeted treatment of colorectal cancer may appear in the future.

## Author contributions

ZY, JW, and XS conceived and designed the study. ZD, RS, and PL selected the studies, collected the data, and drafted the manuscript. FD, JZ, RW, ZL, and MD analyzed the data. All authors interpreted the results and revised the draft manuscript. All authors contributed to the article and approved the submitted version.
